# Real world evidence of improved attention and cognition during physical therapy paired with neuromodulation: a brain vital signs study

**DOI:** 10.3389/fnhum.2023.1209480

**Published:** 2023-06-09

**Authors:** Eric D. Kirby, Christina B. Jones, Shaun D. Fickling, Gabriela Pawlowski, Sonia M. Brodie, Lara A. Boyd, Jan Venter, Nicholas Moser, Sukhvinder Kalsi-Ryan, George Medvedev, Ryan C. N. D’Arcy

**Affiliations:** ^1^BrainNet, Faculty of Applied Sciences, Simon Fraser University, Vancouver, BC, Canada; ^2^Centre for Neurology Studies, HealthTech Connex, Vancouver, BC, Canada; ^3^Brain Behaviour Laboratory, Department of Physical Therapy, Faculty of Medicine, The University of British Columbia, Vancouver, BC, Canada; ^4^Healthcode, Vancouver, BC, Canada; ^5^KITE Research Institute-UHN, Toronto, ON, Canada; ^6^Temerty Faculty of Medicine, Institute of Medical Science, University of Toronto, Toronto, ON, Canada; ^7^Department of Physical Therapy, Rehabilitation Sciences Institute, University of Toronto, Toronto, ON, Canada; ^8^Royal Columbian Hospital, Fraser Health, Vancouver, BC, Canada; ^9^DM Centre for Brain Health, Department of Radiology, The University of British Columbia, Vancouver, BC, Canada

**Keywords:** cranial nerve stimulation, attention, cognition, neuromodulation, neuroplasticity, EEG, ERP

## Abstract

**Background:**

Non-invasive neuromodulation using translingual neurostimulation (TLNS) has been shown to advance rehabilitation outcomes, particularly when paired with physical therapy (PT). Together with motor gains, patient-reported observations of incidental improvements in cognitive function have been noted. Both studies in healthy individuals and case reports in clinical populations have linked TLNS to improvements in attention-related cognitive processes. We investigated if the use of combined TLNS/PT would translate to changes in objective neurophysiological cognitive measures in a real-world clinical sample of patients from two separate rehabilitation clinics.

**Methods:**

Brain vital signs were derived from event-related potentials (ERPs), specifically auditory sensation (N100), basic attention (P300), and cognitive processing (N400). Additional analyses explored the attention-related N200 response given prior evidence of attention effects from TLNS/PT. The real-world patient sample included a diverse clinical group spanning from mild-to-moderate traumatic brain injury (TBI), stroke, Multiple Sclerosis (MS), Parkinson’s Disease (PD), and other neurological conditions. Patient data were also acquired from a standard clinical measure of cognition for comparison.

**Results:**

Results showed significant N100 variation between baseline and endpoint following TLNS/PT treatment, with further examination showing condition-specific significant improvements in attention processing (i.e., N100 and N200). Additionally, CogBAT composite scores increased significantly from baseline to endpoint.

**Discussion:**

The current study highlighted real-world neuromodulation improvements in neurophysiological correlates of attention. Overall, the real-world findings support the concept of neuromodulation-related improvements extending beyond physical therapy to include potential attention benefits for cognitive rehabilitation.

## Introduction

Translingual neurostimulation (TLNS) paired with physical therapy (PT) may facilitate improved rehabilitation outcomes. Clinical adoption of a combined TLNS/PT approach is supported by a growing body of scientific literature that links TLNS/PT to increased neuroplasticity changes in the brain (Danilov et al., 2015; Galea et al., 2017; Hou et al., 2020, 2022). In individuals with mild-to-moderate traumatic brain injury, balance was shown to be both improved and sustained after treatment [TBI; (Tyler et al., 2019; Ptito et al., 2021)]. Recent work in individuals with Multiple Sclerosis [MS; (Tyler et al., 2014)], or stroke has demonstrated the potential for TLNS/PT rehabilitation that spans multiple clinical populations (Danilov et al., 2007; Galea et al., 2017; Leonard et al., 2017; Boughen et al., 2022). Further, within the growing real world clinical experiences, incidental observations of cognitive and mental health improvements have often been reported by clinicians and patients alike (Frehlick et al., 2019; Fickling et al., 2020; Smith et al., 2020).

The Portable Neuromodulation Stimulator (PoNS^®^ ; Helius Medical Technologies: Newtown, PA, USA) is a device that uses the TLNS method to facilitate neurorehabilitation. The PoNS^®^ device involves sequenced non-invasive electrical stimulation applied to the tongue. Trigeminal (CN-V) and facial (CN-VII) cranial nerves have sensory afferents located in the anterior tongue which are engaged by this non-invasive stimulation. This cranial nerve stimulation is thought to co-modulate visual, vestibular, nociceptive and visceral sensory signals via the cranial nerve, brainstem, and cerebellum (Wildenberg et al., 2010, 2011; Danilov et al., 2015). Originally, these neuromodulatory pathways were identified in the facilitation of sensory substitution in balance-impaired and blind individuals (Tyler et al., 2003; Chebat et al., 2007; Danilov et al., 2007; Robinson et al., 2009). When adapted for combined TLNS/PT for balance and gait improvements, clinical studies showed that these improvements are sustained beyond the final stimulation session (Tyler et al., 2003, 2019; Danilov et al., 2007; Paltin et al., 2017).

Beyond sensorimotor effects, our group demonstrated cognitive changes resulting from TLNS that are detected in objective neurophysiological measures using electroencephalography [EEG; (Frehlick et al., 2019; Fickling et al., 2020; Smith et al., 2020)]. EEG is a non-invasive electrophysiological measure of brain activity. Frehlick et al. (2019) showed that 20 min of TLNS alone significantly increased alpha and theta band power along with attention-related EEG microstate activity in healthy individuals. Importantly, the TLNS EEG effects were detectable both immediately and 1-week following the initial stimulation period. Furthermore, the initial stimulation intensity level that an individual received subsequently influenced the response level the following week, which suggested sustained and specific neuromodulation effects in EEG.

To further investigate TLNS effects on cognition in healthy individuals, Smith et al. (2020) conducted a follow up study in which event-related potentials (ERPs) were extracted from EEG recorded before and after participant completed a TLNS stimulation protocol paired with cognitive skills training. This study specifically used the brain vital sign ERP framework (Ghosh Hajra et al., 2016), which uses well-established cognitive ERPs to provide an objective neurophysiological measure of information processing (Gawryluk et al., 2010; Luck, 2014). ERPs are well established quantitative application of EEG. They have been extensively studied since the 1960s and represent the brain’s evoked neural responses to cognitive and sensory stimuli. ERPs have also been shown to have a well-established test–retest reliability (Cassidy et al., 2012). Brain vital sign responses (“brain vital signs”) included the N100 [Auditory sensation (Davis, 1939)], the P300 [Basic attention (Sutton et al., 1967)], and the N400 [Cognitive processing (Kutas and Federmeier, 2011)] ERP components. These components are of particular interest as they are robust to within-subject variance across time (Williams et al., 2005; Cassidy et al., 2012) and can be reliably elicited in healthy individuals. These components are then translated into a 6-score evaluation of brain health (amplitude and latency of all three components). Brain vital sign evaluation can objectively measure brain health and changes over time (Fickling et al., 2019, 2020, 2021a,b; Arvan et al., 2020; Smith et al., 2020; Carrick et al., 2021). It has provided a portable, rapid and automated approach that can be readily integrated with TLNS. When TLNS was paired with cognitive training in healthy individuals, the experimental group showed significant increases in markers of attention and cognitive vigilance relative to controls who had undergone cognitive training without TLNS (Smith et al., 2020).

A recent case report study from our group examined neural and behavioral changes during combined TLNS/PT for increased lower- and upper- limb motor function of an individual with a severe, open TBI survivor who continued to recover over a decade post-injury (D’Arcy et al., 2020). During physical rehabilitation, we also investigated cognitive brain vital signs and showed significant improvements in both attention and, consequently, reported PTSD symptoms (Fickling et al., 2020). Given the above evidence and support for TLNS as a promising mechanism to create change in widespread clinical populations, expanding investigation of cognitive brain vital signs in real-world rehabilitation applications across various neurological disorders represents an important next step.

The objective was to investigate whether combined TLNS/PT resulted in brain vital sign changes from pretreatment baseline to endpoint in a real-world patient sample. Our sample consisted of a diverse group of adults with mild-to-moderate TBI, stroke, Multiple Sclerosis (MS), Parkinson’s Disease (PD) and other conditions. Based on previous work, we hypothesized that combined TLNS/PT would produce ERP attentional effects that could be captured by a brain vital sign approach.

## Materials and methods

**FIGURE 1:**
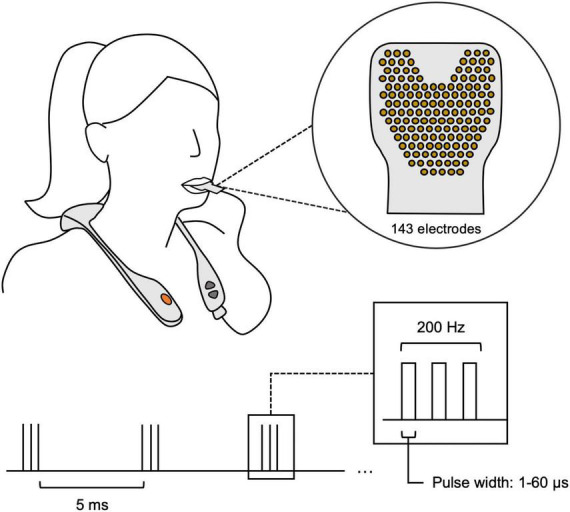
The proper use of the PoNS^®^ neurostimulator device. The device is worn around the neck, with a polyimide substrate including 143 gold-plated electrodes that is placed on the anterior surface of the tongue. The stimulation sequence is briefly portrayed in the lower half, showing 5 ms triplet spacing with a changing pulse width based on the individual’s threshold.

This was a multi-center, retrospective study involving de-identified data from thirty-three (*N* = 33; Table 1) real-world clinical patients with various neurological conditions. The current study pooled a diverse group of TLNS/PT patients (*N* = 33), evaluated with brain vital signs from two large rehabilitation clinics in Canada. All patients underwent baseline assessments, which included a brain vital sign assessment using the NeuroCatch^®^ Platform (NeuroCatch^®^ Inc. Surrey, BC, Canada), and a neuro-cognitive evaluation with the CogBAT. Only a subset of the total patient sample underwent the CogBat (N_COGBAT_ = 19). All patients underwent combined TLNS/PT neurorehabilitation programs focused on balance and gait (Surrey Neuroplasticity Clinic, Vancouver, BC, Canada, *N* = 28 and KITE Clinics–University Health Network, *N* = 5). Specifically, neurorehabilitation programs were the PoNS^®^ Treatment program at the Surrey Neuroplasticity Clinic or the Toronto Rehabilitation Institute. As part of the TLNS/PT program, patients completed physiotherapy focused primarily on improving balance and gait, paired with PoNS^®^ neurostimulation over a 14-week period. Briefly, the TLNS device delivers pulse-width modulated, unbalanced biphasic pulses to the anterior superior surface of the tongue through 143 gold-plated electrodes on a polyimide substrate with a zero net direct current to minimize the potential for tissue irritation. The device delivers triplets of sequenced electrical pulses for a total duration of 20 min per session (Figure 1). The intensity of stimulation was tailored to each individual by altering the pulse width. Intensities were set between the perceptual and discomfort thresholds to ensure patient comfort. PT included four subsections: balance training, gait training, movement training, and breathing and awareness training. The specific tasks during each of these subsections were determined by the treating clinician; individual training exercises varied according to condition and disease severity. However, the program structure and total number of PT hours were the same across all participants. Details of the program have been previously reported by Ptito et al. (2021).

**TABLE 1:** Patient demographic overview.

Neurological condition	Number of patients	Average age (years ± standard deviation)	Gender
TBI	17	49.18 ± 16.9	10 female, 7 male
Stroke	4	73.25 ± 8.7	2 female, 2 male
PD	3	77 ± 5.6	1 female, 1 male
MS	4	66.5 ± 7.3	4 female, 0 male
Other	5	62.2 ± 27.6	3 female, 2 male

**FIGURE 2:**
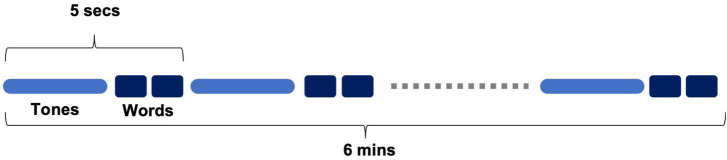
Brief schematic of auditory stimulus sequence consisting of words and tones.

Brain vital signs were recorded using the NeuroCatch^®^ Platform (Version 1.1). NeuroCatch acquired EEG data using an 8-channel g.Nautilus EEG system (g.tec medical engineering, Austria). The data collection involved a 6-min automated stimulus sequence, where participants were presented with auditory tones and prime-target word pairs. The sequence consisted of two types of tones: standard tones (75 dB, 80%) and deviant tones (100 dB, 20%). In addition to the tones, word pairs were interspersed throughout the sequence that were either semantically congruent (“*bread-butter”*) or incongruent (“*bread-window*”) (Figure 2). Standard-deviant tones were used to evoke the N100 and P300, while congruent-incongruent word pairs were used to evoke the N400. Patients were asked to listen attentively, but no active response was required. Patients were also asked to keep their eyes open and fixated on a cross located 2.0 m away (black on white background). EEG data were recorded at 500 Hz and processed to generate brain vital signs for each individual. Ocular artifacts were corrected using adaptive filtering based on electrooculography (EOG) reference inputs; EEG data were subsequently notch filtered (60 Hz), and bandpass filtered (0.5–10 Hz). Processed EEG was then segmented, and baseline corrected (−100–0 ms). Segments exceeding ± 75 uV were rejected and the resulting signals were averaged based on the experimental condition. Data from one patient were excluded due to insufficient signal quality (resulting *N* = 32). Average amplitude windows were determined for each ERP component: N100 component (125–155 ms), P300 component (295–345 ms) and N400 component (465–525 ms) for each individual subject. The windows were chosen based on visual analysis of grouped average component latency onset. Within each window, mean amplitude across the window was calculated and used for statistical analyses. Difference waveforms were calculated by subtracting standard tone waveforms from deviant tone waveforms for each participant. This was included to better visualize the change in the group-level waveform with respect to the deviant tone waveform compared to the standard tone waveform. In addition to the three ERPs contained within the brain vital sign framework, the N200 was characterized to provide an additional measure of attention, with an average amplitude window of (240–270 ms).

The CogBAT (Vienna Test System, Schuhfried, Mödling, Austria) was used as a clinical measure of cognitive status, specific to patients with neurological and/or psychological disorders with the aim to measure cognitive status both as broadly and as time economically as possible. The test uses a package of well-known tests and concepts, such as the Trail Making Test (TMT-L), to measure subdimensions of cognition, such as attention and memory, as well as an overall composite cognition measure (Aschenbrenner et al., 2012).

Statistical analyses were performed with SPSS (IBM Corp., Armonk, NY, USA). To investigate whether the PoNS^®^ Treatment Program produced measurable neurophysiological changes in brain vital signs, repeated measure analysis of variance (rmANOVA) using conservative degrees of freedom (Greenhouse and Geisser, 1959) were conducted on each of the N100 (125–155 ms), P300 (295–345 ms), N400 (465–525 ms) response intervals (N100, P300, and N400). All rmANOVAs investigated differences between condition (deviant, standard—N100 and P300, or incongruent, congruent—N400) and time point (baseline, endpoint). A secondary analysis was conducted to investigate changes to the N200 (240–270 ms) component, as an additional measure of attention. This rmANOVA used the same parameters as the N100 and P300 analyses; investigation of differences between condition (deviant, standard) and time point (baseline, endpoint).

To investigate cognitive testing changes related to the PoNS^®^ Treatment Program, composite CogBAT scores were evaluated for significant differences between baseline and endpoint using a paired student’s *t*-test.

## Results

### Primary analysis

**FIGURE 3:**
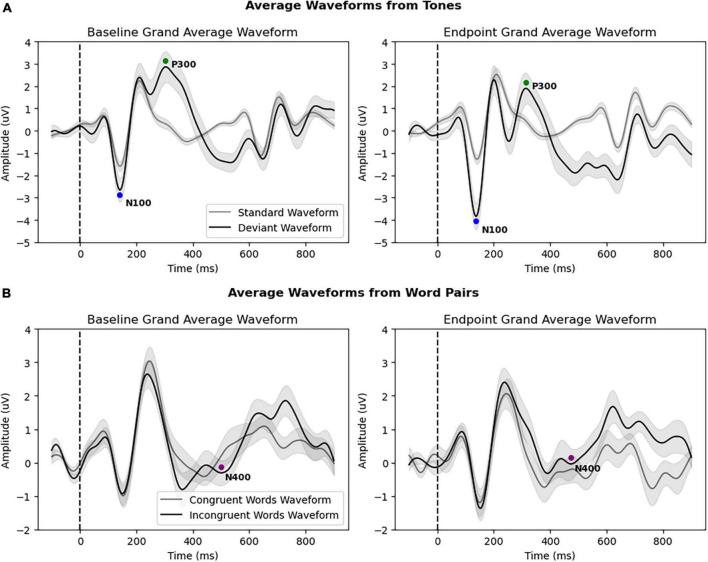
**(A)** Grand average waveforms of all patients (*N* = 32) from standard and deviant tones at baseline and endpoint, with both the N100 and P300 responses. The N100 showed a significant amplitude increase at endpoint. **(B)** Grand average waveforms of all patients (*N* = 32) from congruent and incongruent word pairs at baseline and endpoint, showing the N400 response. All brain vital sign responses are noted with colored circles. The *x*-axis depicts time (in milliseconds), with the stimulus onset denoted by the dotted line (0 ms). The *y*-axis depicts amplitude and polarity (in microvolts).

Figure 3 and Table 2 provide an overview of the N100, P300, and N400 results, showing significant differences in the N100 component between the baseline and endpoint. The rmANOVA of the N100 component resulted in a significant Condition × Timepoint interaction [*F*_(1,31)_ = 5.460, *p* = 0.026, η_p_^2^ = 0.150], which confirmed the difference between standard and deviant conditions changing between baseline and endpoint times. Additionally, a main effect of Condition was present for the N100 [*F*_(1,31)_ = 14.070, *p* < 0.001, η_p_^2^ = 0.312] and P300 [*F*_(1,31)_ = 17.147, *p* < 0.001, η_p_^2^ = 0.356], but not the N400. No significant Timepoint main effect was present for any components in the primary analysis. Lastly, no Condition × Timepoint interactions were found for the P300 or the N400 components.

**TABLE 2:** Primary analysis: Condition × Timepoint interaction.

Component	Window (ms)	Baseline average amplitude (uV)	Endpoint average amplitude (uV)	Condition × Timepoint F-statistic	Condition × Timepoint *p*-value
N100	125–155	−0.74	−2.41	5.460	0.026*
P300	295–345	2.58	1.38	3.843	0.059
N400	465–525	−0.252	0.074	0.767	0.388

Bold indicates *p < 0.05.

### Secondary analysis

**FIGURE 4:**
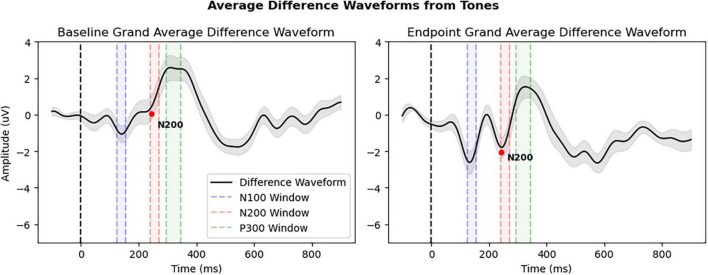
Grand average difference waveforms of all patients (*N* = 32) derived from deviant tone waveform minus standard tone waveform. The N200 peak is denoted by red dot and average amplitude interval windows are highlighted: Window 1: 125–155 ms (N100), Window 2: 240–270 ms (N200), and Window 3: 295–345 ms (P300). All other details as in Figure 3.

Figure 4 and Table 3 provide an overview of the N200 result, showing significant changes in the N200. The rmANOVA of the N200 component confirmed a significant Condition × Timepoint interaction [*F*_(1,31)_ = 8.367, *p* = 0.007, η_p_^2^ = 0.213], as well as a main effect of Timepoint [*F*_(1,31)_ = 4.227, *p* = 0.048, η_p_^2^ = 0.120]. Furthermore, a paired samples *t*-test of CogBAT composite score confirmed a significant difference between baseline and endpoint (Table 3).

**TABLE 3:** Secondary analysis.

Component	Window (ms)	Baseline average (amplitude: uV/Score)	Endpoint average (amplitude: uV/Score)	Condition × Timepoint F-statistic/T-statistic	*p*-value
N200	240–270	0.70	−1.47	8.367	0.007*
CogBAT composite score	N/A	9.74	17.53	2.131	0.047*

Bold indicates *p < 0.05.

## Discussion

The current study highlighted real-world neuromodulation improvements in neurophysiological correlates of attention for patients being treated with combined TLNS/PT across two different clinics. The findings supported the hypothesis that TLNS, delivered through the PoNS^®^, would lead to a significant modulation of the brain’s attentive processes. Specifically, TLNS resulted in a significant Condition × Timepoint effects in average amplitude interval windows encapsulating the N100 and N200 components (Figures 1, 2). The objective neurophysiological changes also corresponded with significant improvements in the composite CogBAT scores from baseline to endpoint.

Cognitive improvements in ERP components related to attention during physical therapy combined with neuromodulation appear to generalize to real world clinical applications across a diverse patient sample. While it is known that TLNS modulates both sensorimotor and vestibular functions via targeted stimulation of cranial afferents facilitating neuroplasticity (Danilov et al., 2015; Galea et al., 2017), the current findings implicated more widespread physiological networks/processes via bottom-up neuromodulation of global brain function (Herrick and Keifer, 1998; Buisseret-Delmas et al., 1999; Marano et al., 2005; Wildenberg et al., 2010, 2011; Brooks et al., 2021). It is important to note that TLNS has previously increased attentive and cognitive processing in healthy individuals (Frehlick et al., 2019; Smith et al., 2020) and in a separate rehabilitation case study of an individual with severe TBI (Fickling et al., 2020), which reinforces the current mixed patient sample results.

Localized to the primary auditory cortex (Zouridakis et al., 1998), the N100 response is associated with early cortical auditory sensory processing (Davis, 1939), which has been linked to selective attention through an increase in neuronal synchronization (Thornton et al., 2007; Delb et al., 2008). Thornton et al. (2007) suggested that the N100 amplitude increases with selective attention as a result of more neurons responding to the stimulus or the same number of neurons responding in greater synchronization. Examination of the waveforms showed that the N100 neuromodulation differences extended to include N200 differences. The N200 also has well-described links to selective attention, particularly cognitive processes of stimulus identification and distinction (Patel and Azzam, 2005).

Danilov and Paltin (2018) previously hypothesized that neuromodulation with trigeminal and cranial nerve stimulation paired with exercise can lead to increased movement, vision, speech, memory, attention, and mood recovery. These effects observed beyond the well documented TLNS-facilitated rehabilitation of balance, posture and gait may be attributed to lasting and cumulative functional, synaptic, and neuronal neuroplastic changes in the brainstem and cerebellum on the cellular and neural network levels (Paltin et al., 2017). Neural activation across various cognitive processing and attention-related systems may coincide with these observed changes. Previous studies using functional magnetic resonance imaging (fMRI) have provided evidence of significant increases in activation within the dorsolateral prefrontal cortex following TLNS (Leonard et al., 2017), implying potential modifications in brain regions associated with attention and working memory performance. However, the precise mechanisms underlying the effects of TLNS have yet to be fully explained. Recent studies have already shown attentive processes are sensitive to neuromodulation, specifically with TLNS (Frehlick et al., 2019; Fickling et al., 2020). Together the current findings point to a conclusion that cognitive improvements in attention arising neuromodulation appear to occur even in a diverse group of patients undergoing rehabilitation treatments for TBI, stroke, MS, PD, and other conditions.

Importantly, the composite CogBAT scores also showed a significant increase (Table 3). While a prior study examined N100 and N200 ERPs in general to CogBAT and did not report a relationship (Şahin et al., 2021), it did not include an active treatment intervention in order to evaluate for improvements over time. Given the current study utilized a diverse sample of patients, future TLNS/PT studies should look at the relationship between cognitive tests, such as the CogBAT, and brain vital signs in more detail.

### Caveats

The study relied on retrospective results of patients undergoing TLNS/PT program. The rehabilitation program was intended as a clinical treatment vs. a controlled study of effects, so all assessments were not completed for every individual, additional disorder etiology could not be determined, and a sham/control group was not available. Scores such as the CogBat were analyzed for only a portion of the individuals who completed the brain vital signs assessments, resulting in a lower number of patients in the subsample. In reporting this data, we aimed to provide a snapshot of the efficacy of the therapeutic use of the TLNS/PT on neurologically impaired patients in the real world. Future studies can further investigate CogBAT sub scores and interactions, along with other behavioral measures, to better characterize the behavioral aspect of neuromodulation as this study focused dominantly on objective physiological changes. Furthermore, the study included a variety of clinical diagnoses as the clients attending the clinics were not limited to a particular neurological condition or disorder. While useful in getting insight into a wide variety of conditions, each subgroup contained smaller samples of patients, with the exception of TBI (*n* = 17). Future studies should include a larger and equal number of patients across different neurological impairments.

## Conclusion

The current study utilized a brain vital signs framework to measure the effects of combined TLNS and PT on cognitive attention within neurologically impaired individuals. Attention improvements were detected in the N100 and N200 responses, suggestive of improvements in selective attention across the diverse real-world patient sample. Our data suggest that combined TLNS/PT may provide additional gains in attention during therapeutic use.

## Data availability statement

The datasets generated and/or analyzed during the current study are not currently publicly available due to intellectual property considerations. Requests to access the datasets should be directed to RD’A.

## Ethics statement

The current study has been approved by the research Ethics Board of Advarra. Data sharing with UHN has been approved by the research ethics board of CAPCR. The patients/participants provided their written informed consent to participate in this study.

## Author contributions

EK contributed with data analysis and manuscript drafting. CJ assisted with data analysis and manuscript drafting. SF, GP, SB, JV, and SK-R contributed with data analysis and manuscript editing. LB and GM contributed with manuscript editing. RD’A assisted in study design, manuscript drafting, and acted as lab PI. All authors contributed to the article and approved the submitted version.
